# Analyzing the Antihyperglycemic Effect of Cissus quadrangularis and Bacopa monnieri on 3T3-L1 Cell Lines

**DOI:** 10.7759/cureus.52661

**Published:** 2024-01-21

**Authors:** Katheeja R, Manish S, Ilangovar IGK, Selvaraj J, Vasugi S

**Affiliations:** 1 Department of Physiology, Saveetha Dental College and Hospitals, Saveetha Institute of Medical and Technical Sciences (SIMATS) Saveetha University, Chennai, IND; 2 Department of Biochemistry, Saveetha Dental College and Hospitals, Saveetha Institute of Medical and Technical Sciences (SIMATS) Saveetha University, Chennai, IND

**Keywords:** antihyperglycemic, 3t3-l1 cell line, gene expression, mtt assay, good health and well-being, bacopa monnieri, cissus quadrangularis

## Abstract

Background

*Cissus quadrangularis* is a perennial shrub of the grape family. Other names for it include devil's backbone, veld grape, and pirandai (Tamil). *Bacopa monnieri*, a perennial plant, is native to wetlands in eastern and southern India. The 3T3-L1 cell line, which was created from 3T3 cells, was used in the scientific study. The current study's purpose is to evaluate the antihyperglycemic benefits of *B. monnieri* and *C. quadrangularis*, which will be added to the current arsenal of efficient herbal hypoglycemic medications.

Aim

To analyze and compare the anti-hyperglycaemic effects of the two plant extracts, *C. quadrangularis* and *B. monnieri* using a 3T3-L1 cell line.

Materials and methods

*C. quadrangularis* seeds were gathered, and extraction was conducted. The *B. monnieri* plant was harvested, and a rotary evaporator was used to extract the flower. Adipocyte cells were obtained from NCCS, Pune. A CO_2_ incubator was used to incubate the cells. The MTT assay and gene expression analysis were done on the cell line samples.

Results

The antihyperglycemic effects of *C. quadrangularis* IRS mRNA levels of 0.7 and AKT mRNA levels of 0.7 are compared to *B. monnieri* IRS1 mRNA levels of 0.6 and AKT mRNA levels of 0.6 to build better diabetic treatments. The antihyperglycemic benefits of *C. quadrangularis* levels of IRS mRNA and AKT mRNA are compared to the influence of B. monnieri IRS1 mRNA and AKT mRNA on the development of better diabetic drugs.

Conclusion

Comparing the effects of *C. quadrangularis* and *B. monnieri* on the 3T3 cell line by gene expression of IRS mRNA and AKT mRNA suggests that the particular AKT downregulation shows that insulin suppresses gluconeogenesis and *C. quadrangularis* inhibits hyperglycemia in 3T3-L1 cells, while research on in vitro rats suggests that *B. monnieri* may minimize the signs and symptoms of diabetes via enhancing IRS1/AKT signaling.

## Introduction

A chronic metabolic condition called diabetes mellitus (DM) involves an unnecessarily increased blood glucose level (hyperglycemia). This hyperglycemia is caused by a lack of insulin secretion or sensitivity to insulin. DM is a feature [1]. There are various types of DM. Insulin resistance and decreased insulin production are the most prevalent types of DM. If left untreated, diabetes has several negative health effects. Hyperosmolar hyperglycemia, diabetic ketoacidosis, and even mortality are examples of acute complications [2]. Namely, numerous pathways leading to an increase in oxidative stress are activated in response to hyperglycemia. The aforementioned include hexosamine biosynthesis pathway activation, enhanced nonenzymatic glycosylation of proteins, and the production of mitochondrial reactive oxygen species (ROS). The overall increased oxidative stress is crucial for the development of major metabolic diseases [3]. Synthetic medications are hazardous and cannot be used continuously; insulin cannot be taken orally. Because of their efficiency, lack of side effects, and affordable price, herbal medications are frequently recommended even when their physiologically active ingredients are unknown [4,5]. As they are derived from natural sources and have fewer adverse effects, medicinal plants are now being used more frequently to treat a variety of health issues [6]. Since ancient times, plants have been the main source of medicine, and many medications have been made either directly or indirectly from various plants [7]. Less than 10% of the over 12,000 plants used for medicine worldwide have been studied from a pharmacological perspective [8].

The 3T3 cell line is a typical fibroblast cell line utilized in a broad range of biomedical research and commercial applications. The 3T3 cell line applications include: researching the role of calcium-mediated actin reset (CaAR) in physiological changes; ascertaining the retroviral construct hTNF's viral titer; and researching how the transient receptor potential vanilloid 3 channel (TRPV3) is activated in adipocyte development [9]. In the last few years, medicinal plants have been used in the world for the treatment of diabetes. Some Indian medicinal plants are most commonly tested for antidiabetic activity and experimentally evaluated. They include *Allium cepa, Alluvium sativum, Aegle marmelos, Aloe vera, Bacopa monnieri, Brassica juncea, Buddleja rapa, Cassia auriculata, Catharanthus roseus, Cajanus cajan, Citrus sinensis, Cissus quadrangularis, Eugenia jambolana, Ficus bengalensis, Gymnema sylvestre, Momordica charantia, Mucuna pruriens, Murraya koeingii, Ocimum sativum, Pterocarpus marsupium, Swertia chirayita, Syzigium cumini, Tinospora cordifolia, Trigonella foenum graceum,* and so on. As herbal drugs have fewer side effects than synthetic allopathic drugs, the World Health Organization (WHO) advises using traditional plants for the treatment of diabetes [10]. The antihyperglycemic activity of the plants is mainly because of their ability to restore the function of pancreatic tissues by causing an increase in insulin output, inhibiting the intestinal absorption of glucose, or facilitating metabolites in insulin-dependent processes. The majority of people benefited from traditional medicines in the management of diabetes [10].

The Vitaceae family, also known as the grape family, included *C. quadrangularis*. It is one of the most widely grown plants in India. *C. quadrangularis *is also known as kandvel in Marathi, harjora in Hindi, pirandai in Tamil, and bone setter in English. They sell this plant all year long. The stems and leaves of *C. quadrangularis* have long been consumed as vegetables [11]. The evergreen climber *C. quadrangularis* grows swiftly and can reach heights of 5 m (1.6 ft) by 5 m (1.6 ft). In the shade, it cannot grow. It enjoys moist or dry soil and can withstand drought. Since ancient times, *C. quadrangularis* has been used as a remedy. It has been used in many Ayurvedic treatments to treat bone fractures and injured tendons and ligaments [12]. Various restorative plants in India have been recognized as having antidiabetic qualities within the conventional restorative framework. Because of their capacity to create from fibroblasts to adipocytes, 3T3-L1 cells are broadly utilized in considering adipogenesis and the natural chemistry of adipocytes [13]. Ayurvedic professionals have been utilizing the conventional herb *B. monnieri* for thousands of years as a narcotic, pain relieving, antipyretic, anti-inflammatory, and antiepileptic. Various research facilities have carefully investigated the plant, plant extricate, and extricated bacosides (the essential dynamic components) for their neuropharmacological impacts. There are a huge number of considerations accessible affirming the nootropic activity of these substances [14]. *B. monnieri *Wettst, often known as brahmi, may be an inching, bitter-tasting plant that flourishes in sodden, mucky environments. It has a place in the *Scrophulariaceae *family and is regularly utilized within the conventional Ayurvedic restorative framework as a diuretic, cardiotonic, nerve tonic, and as a cure for epilepsy, sleep deprivation, asthma, and stiffness. Furthermore, various considerations uncovered that this plant extricate contains antioxidant, upper, and anxiolytic exercises [15]. The essential work of adipocyte cells is to store the extra vitality obtained from nourishment utilization for afterward utilization by cells and tissues. In any case, unhealthy lifestyle choices, such as diligent indulging and dormancy, have appeared to essentially contribute to the improvement of fat tissue. The objective of the study is to assess the antihyperglycemic movement of *C. quadrangularis* and *B. monnieri* against 3T3-L1 cell lines [16].

## Materials and methods

Chemicals

From Gibco, Canada, we obtained trypsin-ethylenediaminetetraacetic acid (EDTA), fetal bovine serum (FBS), antibiotics and antimycotics, Dulbecco’s Modified Eagle Medium (DMEM), and phosphate-buffered saline (PBS). The real-time polymerase chain reaction (RT-PCR) kit (MESA Green) and JC-1 are bought from Invitrogen in the USA. All of the compounds were of the highest purity and analytical quality [17].

Extract preparation

**Figure 1:**
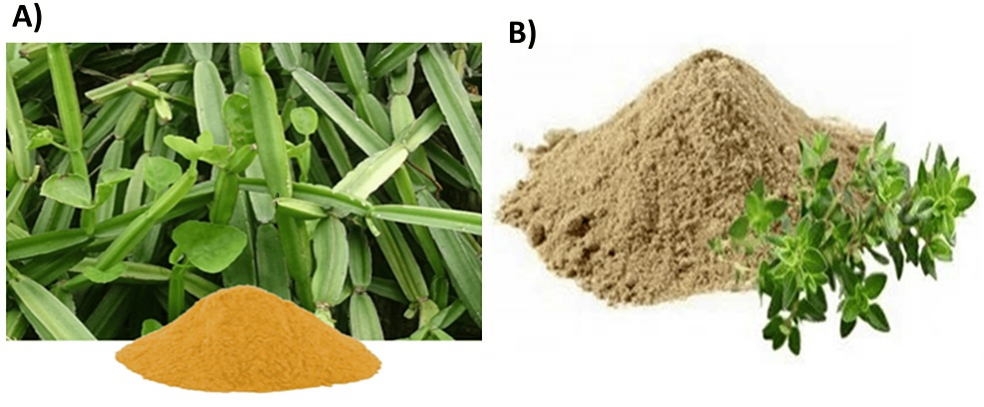
(A) Extract of C. quadrangularis and (B) extract of B. monnieri

The powder was made by Soxhlet extraction of *C. quadrangularis* (Figure 1(A)) and *B. monnieri* (Figure 1(B)) in 70% ethanol. A viscous mass was produced by evaporating the solvent from the extract using a Rotary evaporate apparatus at low pressure, and it was then kept at 4°C until it was needed. The extraction process was completed using filter paper [18].

Collection and cultivation of adipocyte cell lines

The National Centre for Cell Science, Pune, India, supplied the adipocyte cell line, which was then cultivated in compliance with the guidelines. Adipocyte cell lines were, in essence, cultured in Dulbecco's Modified Eagle Medium containing 10% fetal bovine serum at 37°C and 5% CO_2_ [19].

Cytotoxicity studies 

In 96-well plates, 5x10^5^ cells/well were used to seed 3T3-L1 cell lines, which were then allowed to adhere to the well for the duration of the night. Following incubation, the grown cells were stimulated in triplicate using different concentrations of *B. monnieri* extracts. They were then kept in an incubator with 5% humidified CO_2_ at 37°C for a whole day. Each well was then filled with MTT test, and the incubation was maintained at 37°C for an additional 4 h. The cells were once more suspended in 200 µl dimethyl sulfoxide to dissolve the formazan that was formed by the MTT (3-(4,5-dimethylthiazol-2-yl)-2,5 diphenyl tetrazolium bromide) test. To determine the solution's optical density at 570 nm, the spectrophotometer was calibrated. Three independent repetitions of the trials were conducted. For every set of replicates, the mean optical density (OD) ± standard deviation was determined. The complete process was conducted three times. The inhibitory rate of cell growth was calculated using the equation: % growth inhibition = (1 - OD extract treated)/OD negative control x 100 [20].

Gene expression analysis

Using RT-PCR, the levels of mRNA expression were ascertained. Tri Reagent (Sigma) was used to isolate total RNA. Total RNA (2 g) from each sample was reverse transcribed using a commercial Superscript III first-strand cDNA synthesis kit (Invitrogen, USA) in compliance with the manufacturer's instructions. The MX3000p PCR machine from Stratagene, Europe, was used for real-time polymerase chain reaction. The reaction was conducted using the MESA Green PCR master mix, which contains all of the PCR components, including SYBR green dye. In the US, Eurogentec was established. Melting curve analysis was used to determine the specificity of the amplified product for each primer combination. After processing the data using the comparative CT methodology in CFX Manager Version 2.1 (Bio-Rad, USA), the fold change was determined by utilizing the 2CT method proposed by Schnittger and Livak [21].

Statistical analysis

The means of standard deviation were used to display the findings of three separate experiments. Every study was conducted twice. The statistical analysis used a one-way analysis of variance, and a result was considered statistically significant if its P value was 0.05 or above [22].

## Results

IRS mRNA gene expression

**Figure 2:**
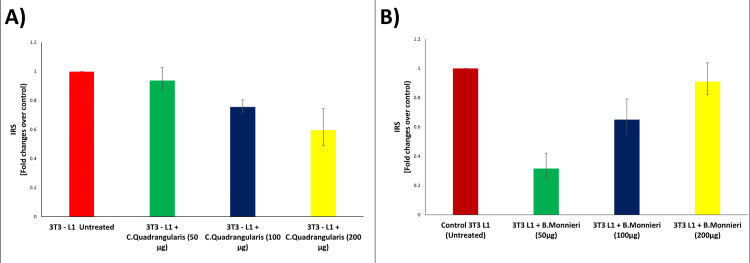
(A) IRS mRNA expression of C. quadrangularis and (B) IRS mRNA expression of B. monnieri IRS: insulin receptor substrate; mRNA: messenger ribonucleic acid

The graph depicts insulin receptor substrate (IRS) mRNA gene expression in *C. quadrandularis *(Figure 2(A)) and IRS mRNA gene expression in *B. monnieri *(Figure 2(B)). The mean value of the IRS fold change in untreated cells in *C. quadrangularis* and *B. monnieri* was determined to be 1.0. The IRS fold change in cells treated with 50 μg of *C. quadrangularis* extract is 0.937, while in *B. monnieri* it is 0.316. The IRS fold change in 100 μg of *C. quadrangularis* extract was 0.755, while it was 0.65 in 100 μg of *B. monnieri* extract. The IRS1 fold change after 200 μg of *C. quadrangilaris* extract is 0.595, and the IRS1 fold change after* B. monnieri* extract is 0.91, which is nearly identical to the initial untreated cells, demonstrating the antihyperglycemic activity of *B. monnieri *plant extract compared to *C. quadrangularis.*

AKT mRNA gene expression

**Figure 3:**
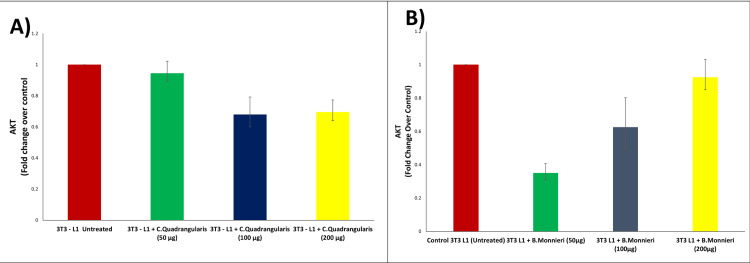
(A) AKT mRNA expression of C. quadrangularis and (B) AKT mRNA expression of B. monnieri AKT: Ak strain transforming; mRNA: messenger ribonucleic acid

The graph displays Ak strain transforming (AKT) mRNA gene expression in *C. quadrandularis *(Figure 3(A)) and AKT mRNA gene expression in *B. monnieri *(Figure 3(B)). The mean value of the AKT fold change in untreated cells was determined to be 1.0 in *C. quadrangularis* and *B. monnieri.* The mean value of the AKT fold change in cells treated with 50 μg of *C. quadrangularis* was 0.94, whereas the mean value of the AKT fold change in cells treated with *B. monnieri* extract was 0.35. The mean value of AKT fold change after 100 μg is 0.679 in *C. quadrangularis* extract and 0.625 in *B. monnieri extract*. The mean value of the AKT fold change after 200 μg of *C. quadrangularis* extract was found to be 0.695 and *B. monnieri *extract was found to be 0.925, which is practically identical to the original untreated cells, demonstrating the antihyperglycemic action of the *B. monnieri* plant extract compared to *C. quadrangularis*. 

## Discussion

The essential insulin downstream signaling pathway known as PI3K/Akt regulates a wide range of physiological and pathological processes, including cell formation, survival, and glucose metabolism. Insulin predominantly interacts with insulin receptors in the liver, skeletal muscles, and adipose tissues via activating IRS-1 tyrosine phosphorylation. Following that, phosphorylated IRS-1 binds to p85, a PI3K regulatory component, prompting AKT and GSK3 to become phosphorylated. GSK3 may then influence how the body consumes glucose by regulating glycogen production, gluconeogenesis, and glucose transport [23]. Several physiological processes, including glucose transport, glycogen synthesis, DNA synthesis, antiapoptotic activity, and cell proliferation, are said to be significantly influenced by the AKT and IRS proteins. As a result, extensive research has concentrated on other areas of cell biology, tissue formation, and other areas outside of the metabolic effects of insulin [24].

As the quantity of 3T3-L1 cells treated with *C. quadrangularis* and *B. monnieri *grew, the expression of the insulin receptor substrate was shown to be decreasing. When compared to the control, there was a noticeable decrease. AKT is a downstream insulin signaling effector [25]. Using 3T3-L1 cells, *C. quadrangularis* demonstrated that proliferation is reduced as the concentration rises. This demonstrates that the extract of *C. quadrangularis* may contain strong bioactive substances that inhibit hyperglycaemic activity [26]. The WSSP extract's bioactive components, including anthocyanins and alkaloids, are thought to be responsible for the substance's antidiabetic effects [27]. The mean value of the IRS1 fold change in the untreated cells was noted to be 1.0. The mean value of the IRS1 fold change in the cells treated with high glucose (16.5 mmol/l) was noted to be 0.316. The mean value of the IRS1 fold change after the addition of 50 μg of *B. monnieri *extract was noted to be 0.65. The mean value of the IRS1 fold change after the addition of 100 μg of *B. monnieri *extract was noted to be 0.91, which is almost similar to that of the initial untreated cells, hence proving the antihyperglycemic activity of the *B. monnieri* plant extract [28]. The mean value of the AKT fold change in the untreated cells was noted to be 1.0. The mean value of the AKT fold change in the cells treated with high glucose (16.5 mmol/l) was noted to be 0.35. The mean value of the AKT fold change after adding 50 μg of *B. monnieri *extract was noted to be 0.625. The mean value of the AKT fold change after the addition of 100 μg of *B. monnieri *extract was noted to be 0.925, which is almost similar to that of the initial untreated cells, hence proving the antihyperglycemic activity of the *B. monnieri *plant extract [29]. *C. quadrangularis* has downregulated his expression in AKT mRNA action, as we see with increased concentrations from 50 µg to 200 µg.

The percentage control of cytotoxicity of the untreated 3T3 L1 cell line was noted to be 100%. After the addition of 16.5 mmol/L glucose, the percentage control of cytotoxicity decreased to 87.5%. The percentage concentration after the addition of 50 μg of *B. monnieri* stayed the same. After the addition of 100 μg of *B. monnieri*, the noted percentage was 82%. From previous research, it was found that the ethanolic extract of *B. monnieri* stopped the diabetic rats from losing weight and brought them back to almost normal. In vitro, the extract boosted peripheral glucose uptake in diabetic rats' diaphragms, which is comparable to insulin's effects. Only oral hypoglycemic medications and insulin have been used to treat diabetes mellitus, with the former being known to have substantial side effects. As a result, the extract may have insulin-like activity, and its ability to lower blood sugar levels may be a result of increased consumption of glucose by the peripheral nervous system and defense against oxidative stress in alloxanized diabetes. In alloxan-induced hyperglycemic rats, the extract significantly reduced blood glucose levels compared to the controls [30].

Limitations

The extract prepared from two known groups of plants has some effective function in various dosages but does not provide complete gene expression. The time of reaction has been slow, and we cannot find the effect in the fixed time. Some effects in gene expression cannot be effective for the results as expected. For the time being, the antidiabetic activity of *C. quadrangularis* and *B. monnieri* has only been studied in vitro, but further study with animal in vivo models is possible. Following in vivo testing, it could be used as an effective therapeutic antidiabetic medicine in future investigations.

## Conclusions

The unique expression of the genes involved in gluconeogenesis is revealed by the effects of *C. quadrangularis* on IRS insulin resistance, and specific AKT downregulation indicates that insulin inhibits gluconeogenesis. *C. quadrangularis* prevents hyperglycemia in 3T3-L1 cells. It has been demonstrated that *B. monnieri *lowers blood sugar levels, and the effects of *C. quadrangular* IRS mRNA levels of 0.7 and AKT mRNA levels of 0.7 are compared to *B. monnieri* IRS1 mRNA levels of 0.6 and AKT mRNA levels of 0.6 to design better diabetic treatments. The AKT and IRS1 genes were successfully expressed in the 3T3 L1 cell line. This research suggests that *B. monnieri* has an effective hypoglycemic effect in treating the signs and symptoms of diabetes. Finally, we conclude that *B. monnieri* has a stronger antihyperglycemic effect than *C. quadrangularis*.
